# Morphological and Biomechanical Differences in the Elastase and AngII *apoE* ^*−/−*^ Rodent Models of Abdominal Aortic Aneurysms

**DOI:** 10.1155/2015/413189

**Published:** 2015-05-03

**Authors:** Evan H. Phillips, Alexa A. Yrineo, Hilary D. Schroeder, Katherine E. Wilson, Ji-Xin Cheng, Craig J. Goergen

**Affiliations:** ^1^Weldon School of Biomedical Engineering, Purdue University, 206 S. Martin Jischke Drive, West Lafayette, IN 47907, USA; ^2^Department of Biomedical Engineering, John A. White, Jr. Engineering Hall, 790 W. Dickson Street, Suite 120, Fayetteville, AR 72701, USA

## Abstract

An abdominal aortic aneurysm (AAA) is a potentially fatal cardiovascular disease with multifactorial development and progression. Two preclinical models of the disease (elastase perfusion and angiotensin II infusion in apolipoprotein-E-deficient animals) have been developed to study the disease during its initiation and progression. To date, most studies have used *ex vivo* methods to examine disease characteristics such as expanded aortic diameter or analytic methods to look at circulating biomarkers. Herein, we provide evidence from *in vivo* ultrasound studies of the temporal changes occurring in biomechanical parameters and macromolecules of the aortic wall in each model. We present findings from 28-day studies in elastase-perfused rats and AngII *apoE^−/−^* mice. While each model develops AAAs specific to their induction method, they both share characteristics with human aneurysms, such as marked changes in vessel strain and blood flow velocity. Histology and nonlinear microscopy confirmed that both elastin and collagen, both important extracellular matrix molecules, are similarly affected in their levels and spatial distribution. Future studies could make use of the differences between these models in order to investigate mechanisms of disease progression or evaluate potential AAA treatments.

## 1. Introduction

Abdominal aortic aneurysms (AAAs) are a potentially life-threatening disease, generally defined as a focal dilation of the aorta to 50% above normal [1]. AAAs accounted for approximately 11,000 deaths in the United States in 2009 and had a prevalence rate of 12.5% for men 75 to 84 years of age in 2006 [2]. The maximum aortic diameter and rate of vessel expansion in patients are the most important clinical measures in monitoring AAA progression. However, the unpredictable nature of aortic aneurysm rupture has led to the active investigation of potential biomarkers and imaging strategies that can be used to better predict the risk of rupture or improve treatments [3].

Preclinical animal research is important to better understand aneurysm pathophysiology, identify biomarkers, and develop treatment strategies that can be translated to the clinic. Researchers have used two common small animal models in order to investigate AAA disease progression [4, 5] or experimental treatment effects [6–8]. In one model, pressurized intra-aortic elastase perfusion in the infrarenal aorta of rats [9, 10] or mice [11] induces mechanical expansion of the vessel. The perfused region further expands as macrophages infiltrate the vessel wall and matrix proteins in the vessel wall remodel [9, 11]. With the second model, subcutaneous systemic infusion of angiotensin II (AngII) in genetically modified hyperlipidemic mice (*apoE* ^*−/−*^) leads to aortic dissection and expansion of the suprarenal aorta [5, 12]. Due to these induction mechanisms, there are fundamental differences between each model, but they both mimic portions of the human disease [13]. Given the multifactorial development of AAAs* in vivo*, studies that rigorously quantify and monitor progression in multiple disease models are of high value in contributing to our understanding of vessel dynamics and AAA heterogeneity.

Noninvasive imaging technologies used in the clinic, such as magnetic resonance imaging (MRI) and ultrasound (US), are now being used in preclinical AAA research [14]. Research groups have used MRI and US in longitudinal studies to monitor changes in aortic diameter [15–18] and vessel motion [19–21]. Without the aid of these noninvasive imaging techniques, studies are typically limited to a single experimental time point per animal. This increases the total number of animals needed and prevents studying further disease progression once the animal is sacrificed. Furthermore, dynamic* in vivo* measurements such as vessel motion and blood flow are not possible.

In this paper, we present the results of longitudinal ultrasound studies using the elastase and AngII* apoE* ^*−/−*^ animal models to compare the changes in four important anatomic and biomechanical parameters: aneurysm diameter, aneurysm volume, circumferential cyclic strain, and blood flow velocity. Additionally, we show results from semiquantitative AAA histological tissue analysis* ex vivo* to detect changes in elastin and collagen content for each model. Finally, we demonstrate the use of label-free nonlinear optical microscopy as a surrogate for histology. Our findings suggest that the biomechanical parameters measured here change drastically due to vessel wall remodeling as the aortic diameter and volume increase. By measuring these parameters in individual AAA rats and mice during disease progression, further* in vivo* studies investigating potential therapeutics can be performed.

## 2. Materials and Methods

### 2.1. Animal Care and Maintenance

Male Sprague Dawley rats (281–347 g; 10-11 weeks old) from Harlan Laboratories (Indianapolis, IN) and male and female apolipoprotein-E-deficient mice (20–36 g; 17 ± 7.8 weeks old; B6.129P2-*Apoe* ^*tm1Unc*^/J strain) from The Jackson Laboratory (Bar Harbor, ME) were used for elastase perfusion and osmotic pump studies, respectively. All animals were allowed free access to standard rodent chow and water. We recorded animal weights prior to surgery, after surgery, and on subsequent study days. All studies lasted up to 29 days postsurgery at which point animals were euthanized (isoflurane and carbon dioxide asphyxiation). The Purdue Animal Care and Use Committee approved all experiments.

### 2.2. Elastase Perfusion Surgery in Rats

We induced AAAs in Sprague Dawley rats (*n* = 11) using an intra-aortic elastase perfusion procedure described previously [9]. Briefly, the infrarenal aorta was surgically exposed in an anesthetized rat (2% isoflurane) and proximal and distal aortic sites were temporarily ligated with 6-0 silk sutures. Type I porcine pancreatic elastase solution (E1250, Lot number SLBD0685V; Sigma Aldrich, St. Louis, MO) was pressure-perfused (100 mm Hg) for 30 minutes into the ligated aorta through an aortotomy proximal to the aortic bifurcation. We tested both low (0.44–0.48 U/mL; *n* = 6) and high (25 U/mL; *n* = 5) elastase solutions.

### 2.3. Implantations of Angiotensin II-Loaded Miniosmotic Pumps in Mice

We used a second AAA model [12] in which continuous angiotensin II (AngII) infusion in apolipoprotein-E-deficient (*apoE* ^*−/−*^) mice induces suprarenal AAA formation typically between 3 and 14 days after implantation of miniosmotic pumps (ALZET Model 2004; DURECT Corporation, Cupertino, CA). AngII powder (MW: 1046.19; Bachem, Torrance, CA) was solubilized in 0.9% sodium chloride and loaded into miniosmotic pumps for systemic hormone delivery (1000 ng/kg/min infusion rate and 28-day duration) following subcutaneous implantation in the dorsum of mice (*n* = 7).

### 2.4. Small Animal Ultrasound Imaging

We performed* in vivo* ultrasonography of rats and mice kept unconscious under inhalant anesthesia (1–2.5% isoflurane). Prior to imaging, animals were positioned supine on an adjustable heated stage and sterile eye lubricant was applied to each eye. We noninvasively monitored heart and respiration rate through stage electrodes and body temperature using a rectal probe. Hair on the ventral abdomen was removed with a depilatory cream and warm transmission ultrasound gel was applied on the exposed skin surface.

For detection and longitudinal monitoring of aortic diameter, aortic volume, vessel wall motion, and blood flow dynamics, we used a high-resolution small animal ultrasound system (Vevo2100 Imaging System; VisualSonics, Toronto, ON, Canada). We acquired transaxial and longitudinal ultrasound data prior to surgeries (day 0) and at days 3, 7, 14, 21, and 28 postsurgery using VisualSonics linear array transducers MS550D (25–55 MHz, 40 MHz center frequency) and MS250D (13–24 MHz, 21 MHz center frequency) in mice and rats, respectively. The transducer was positioned perpendicular to the animal and held in contact with the gel during imaging. We adjusted the angle of the stage as necessary to allow optimal visualization of the aorta in the long and short axes. We used anatomical landmarks, such as the inferior vena cava, renal veins, and aortic bifurcation, for orientation and the presence of vessel wall motion for discriminating arterial from venous flow.

High-resolution, two-dimensional cine loops in brightness mode (B-mode; 300 frames), motion mode (M-mode; five-second acquisition), pulsed wave (PW), and color Doppler (five-second acquisitions) were acquired in aortic regions with and without vessel expansion. We adjusted the transducer beam angle and PW Doppler angle (30–60 degrees from the vertical) in order to accurately detect the magnitude and direction of blood flow in the selected aortic area. Additionally, we used respiration and cardiac gating for transaxial B-mode volumes (0.19 mm step size) of the infrarenal aorta of rats (3 cm scan distance) and the suprarenal aorta of mice (1.5 cm scan distance).

### 2.5. Blood Pressure Measurements

We measured blood pressures from the tails of conscious* apoE* ^*−/−*^ mice in the study prior to pump implantation and on days 3, 14, and 28 after implantation. Prior to implantation, mice were acclimated to restraint holders and the tail cuffs used for taking blood pressure measurements (CODA 2 Channel Standard, Kent Scientific, Torrington, CT).

### 2.6. Ultrasound Analysis

We analyzed all ultrasound data (days 0, 3, 7, 14, 21, and 28) from rats and mice with Vevo2100 software (VisualSonics) to determine maximum aortic diameter, AAA true and false lumen volumes, circumferential cyclic strain, and mean blood flow velocity. All measurements on cine loops were made in quintuplicate and averaged.

B-mode volumes of the abdominal aorta were segmented in a semiautomated fashion by drawing serial contours (step size: 760 *μ*m and smaller). The length and location of maximum AAA diameter in relation to the right renal artery in mice or the aortic bifurcation in rats were used to guide volume measurements at earlier time points. In other words, earlier day volumes were segmented over the same anatomical length and started at the same anatomical position. In the AngII* apoE* ^*−/−*^ mice, volumes of both the AAA lumen and total AAA (including the vessel wall) were segmented individually. False lumen volumes were calculated as the difference between the total AAA and the true lumen.

We further calculated effective maximum diameter from these segmented volumes. We identified the slice showing maximal aortic cross-sectional area and calculated effective maximum diameter using the equation for area of a circle and the measured area. In three instances, aortic diameter measurements from long-axis B-mode were substituted for the displayed effective maximum diameter measurements, as the collected volumes were not clear enough to provide effective maximum diameter values.

Aortic diameters in areas of maximal vessel expansion as seen in long axis were also measured from high-temporal resolution B-mode cine loops (50 frames/sec for MS250D; 233 frames/sec for MS550D). We employed a straight-line distance measurement tool to calculate the length from the posterior to anterior vessel wall at peak systole and end diastole, corresponding to systolic (*D*_*S*_) and diastolic (*D*_*D*_) diameters. Mean aortic diameter (*D*_*M*_) for a given animal on a given day was calculated as follows:

(1) DM=13DS−DD+DD.

We calculated circumferential cyclic strain values from M-mode diameter measurements in areas showing maximum vessel expansion seen in long axis. The distance between two lines of strong specular reflection seen in the anterior and posterior vessel walls was used to determine maximum and minimum vessel wall displacement. These respective values correspond to the timing of peak systole and end diastole. In order to calculate Green-Lagrange circumferential cyclic strain, the following formula was used [20, 22]:

(2) $$\begin{array}{c} \frac{1}{2}\left[{\left(\frac{D_{S}}{D_{D}}\right)}^{2}-1\right]\times 100\%. \end{array}$$

Curvature in the suprarenal aorta, however, requires a geometric correction to be applied to the initial diameter (*D*_*i*_) measurements made for the AngII* apoE* ^*−/−*^ mice. The angle (*θ*) between vertical and a line segment perpendicular to anterior and posterior vessel walls in the AAA was determined. Prior to the circumferential cyclic strain calculation above, the following equation was used to calculate geometrically corrected diameter (*D*_*c*_) values:

(3) Dc=Di∗cos⁡θ.

Mean blood flow velocity values were calculated from velocity waveforms on PW Doppler images. A velocity waveform area analysis tool was used to calculate this value over individual cardiac cycles displayed on a velocity/time plot.

### 2.7. Animal Perfusion and Dissection

After euthanasia, animals underwent a series of pressure perfusions through the left ventricle (transcardial perfusion). We successively perfused 0.9% sodium chloride (to flush the systemic vasculature), 4% paraformaldehyde (to fix the aorta), and 1% molten agarose (to preserve vessel patency upon cooling) using a syringe pump connected to a feeding needle or polyethylene tube (0.64 mm outer diameter) inserted in the left ventricle. The heart, aorta, and kidneys were later dissected from the animal and fixed in 4% paraformaldehyde for 24 hours. Aortic tissue was kept in phosphate-buffered saline until gross vessel dissection (four 2-3 mm-long cross-sections) in proximal, aneurysmal, and distal areas and then held in 70% alcohol until processing.

### 2.8. Aortic Tissue Histology and Staining

Aortic segments were paraffin-embedded, thin-sectioned (5 *μ*m), and adjacent sections stained with hematoxylin and eosin (H&E), Verhoeff Van-Gieson (VVG)/hematoxylin, and Masson's trichrome (MTC) as per standard protocols. The latter two stains were used to differentiate elastin bands (black) and collagen fibers (blue), respectively, from surrounding structures. Stained tissue sections were scanned at 40x magnification.

### 2.9. Nonlinear Optical Microscopy

H&E-stained aortic sections were imaged using two nonlinear optical (NLO) microscopy techniques, two-photon excitation fluorescence (TPEF), and second harmonic generation (SHG) for label-free imaging of endogenous macromolecules. A custom-built femtosecond laser system (140 fs pulse width; 80 MHz repetition rate) was used for simultaneous TPEF/SHG imaging at 800 nm excitation (Chameleon Ultra I; Coherent, Santa Clara, CA). A tightly focused beam (39 mW average power) was directed through a 20x air or 60x water immersion objective on an inverted microscope (IX70, Olympus, Melville, NY). FV300 software (Olympus, Melville, NY) was used for image capture and thresholding of forward-emitted SHG and back-reflected TPEF autofluorescence signals detected by separate photomultiplier tubes (PMTs). A dichroic mirror (550dcxr, Chroma Technologies, Rockingham, VT) separated the excitation beam from back-reflected TPEF signal. Band-pass filters were chosen according to a previous publication focused on label-free imaging of collagen and elastin in swine arteries [23].

### 2.10. Image Processing and Semiquantitative Analysis

VVG images of aneurysmal and control (i.e., proximal or distal) aortic sections from the suprarenal (AngII* apoE* ^*−/−*^ mice) or infrarenal (elastase rats) aortas were analyzed using a semiautomated thresholding technique described previously [20] to acquire semiquantitative measures of elastin area. The thresholding technique was further modified to generate binary images of collagen pixels from corresponding MTC images. Hand-selected regions of interest were placed in three locations on the aortic adventitia of each image to measure the density of pixels corresponding to collagen and averaged.

### 2.11. Statistical Analysis

We used paired *t*-tests and two-sample *t*-tests in order to assess statistical significance of a parameter between two time points or between AAA and a healthy region. Additionally, we used one-way analysis of variance to test for significant differences in mean systolic or diastolic blood pressure at multiple times postimplantation as compared to baseline (Dunnett's test). Significance at *α* = 0.05 was used for all tests.

## 3. Results

### 3.1. Small Animal Ultrasonography of AAAs Enables Longitudinal Monitoring of Aortic Diameter Expansion and Volume Growth over Time

**Figure 1:**
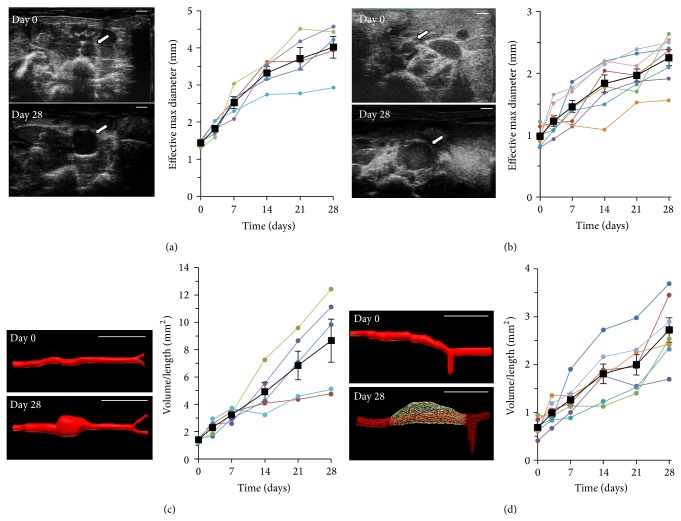
AAA diameter and volume increases over 28 days. Representative B-mode images (maximum aortic cross-section), aortic volumes (days 0 and 28), and longitudinal measurements of effective maximum diameter ((a), (b)) and volume/length ((c), (d)) in high-concentration-elastase rats ((a), (c)) and AngII* apoE* ^*−/−*^ mice ((b), (d)). Arrows point to abdominal aorta in cross-section. Volume segmentations: 30 mm in rats and 15 mm in mice (0.19 mm step size). Scale bars: 2 mm (a), 1 mm (b), 10 mm (c), and 5 mm (d).

Two-dimensional and three-dimensional B-mode scans enabled us to comprehensively image aortas in AngII* apoE* ^*−/−*^ mice and high-concentration-elastase rats in the short- (transaxial) and long-axis (sagittal) orientations.  Figure 1 shows representative B-mode images of the planes of section where the maximal aortic cross-sectional area was found at days 0 and 28 for the high-concentration-elastase rat (Figure 1(a)) and AngII* apoE* ^*−/−*^ mouse (Figure 1(b)) studies. During the course of the studies, we clearly identified areas of local aortic expansion (arrows in Figures 1(a) and 1(b) for day 28) and tracked this increase in effective maximum diameter (Figures 1(a) and 1(b), graphs). Proximal and distal aortic areas with normal vessel morphology were imaged and showed no overall increase in diameter (data not shown).

Table 1 shows the average increases in effective maximum diameter by the end of the study. Over 28 days, AAAs in high-concentration-elastase rats grew 181 ± 13.8% relative to baseline (Figure 1(a)), and those in AngII* apoE* ^*−/−*^ mice grew by 126 ± 10.5% (Figure 1(b)). High-concentration-elastase rats exhibited obvious formation of AAAs postsurgery and a progressive increase (94 *μ*m/day on average) in effective maximum diameter (Figure 1(a)) thereafter (1.78–3.39 mm larger at day 28 than at baseline, 95% CI; *P* < 0.05). Low-concentration-elastase rats, however, did not show significant vessel expansion by ultrasound or a significant change in effective maximum diameter over 28 days (Table 1). Given the spontaneous development of initial aortic expansion in the AngII* apoE* ^*−/−*^ model (between days 3–14), maximum aortic diameter was 86.7 ± 8.36% larger at day 14 imaging on average (Figure 1(b)) with a growth rate of 198 *μ*m/day thereafter until day 28.

We further measured AAA volumes from segmented three-dimensional B-mode scans (Table 1). In high-concentration-elastase rats, there was a noticeable volume expansion in the perfused region as compared to low-concentration-elastase rats. Volume/length values for the high-concentration-elastase rats increased by 516 ± 96.0% after 28 days (Figure 1(c)). In AngII* apoE* ^*−/−*^ mice, we observed saccular AAAs with volume/length values increasing by 297 ± 42.5% for AAA total volumes (Figure 1(d)). False lumens were identified at day 3 (*n* = 2), day 7 (*n* = 2), and day 14 (*n* = 3) imaging. Between days 3 and 28, these volumes increased in size by 774 ± 512% overall. As well, we found that there was a significant change in the percentage of AAA volume attributable to the false lumen (20.8 ± 11.8% versus 57.1 ± 10.2%, resp.; *P* < 0.001).

### 3.2. Circumferential Cyclic Strain Rapidly Decreases in AAAs

**Figure 2:**
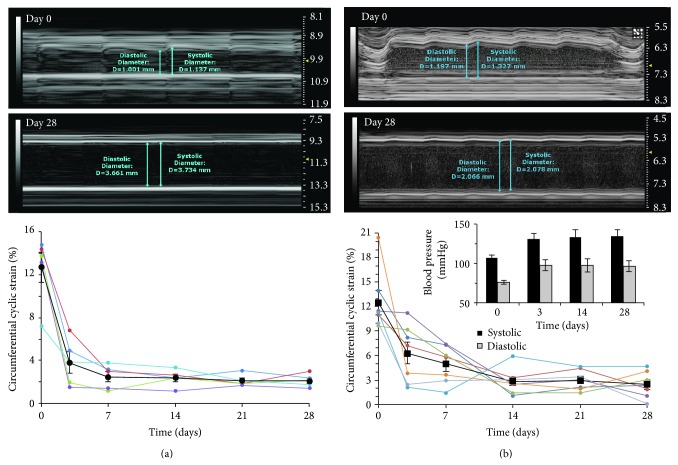
Circumferential cyclic strain decreases over 28 days. Representative long-axis M-mode tracings (days 0 and 28) and longitudinal measurements of circumferential cyclic strain at site of AAA for high-concentration-elastase rats (a) and AngII* apoE* ^*−/−*^ mice (b). Inset in (b): longitudinal measurements of systolic and diastolic blood pressure in AngII* apoE* ^*−/−*^ mice.

We observed abrupt changes in vessel wall displacement and Green-Lagrange circumferential cyclic strain values in high-concentration-elastase rats (Figure 2(a), graph) and AngII* apoE* ^*−/−*^ mice (Figure 2(b), graph) at day 3 relative to baseline (−69.9 ± 26.2%, *P* = 0.0048; −49.7 ± 11.9%, *P* = 0.026, resp.). These values decreased in the suprarenal aortas of some mice where vessel motion had diminished, but initial aortic expansion had not yet occurred. Between days 3 and 28, we measured continued reductions in these values (−43.9 ± 28.3%, *P* = 0.0048; −60.7 ± 19.6%, *P* = 0.054, resp.) and visualized stiffer vessel walls in AAAs (see Supplementary Videos 1 and 2 of the Supplementary Material available online at http://dx.doi.org/10.1155/2015/413189). In low-concentration-elastase rats, we also found that the Green-Lagrange circumferential cyclic strain values significantly decreased over 28 days (Table 1) likely due to mechanical damage from the pressurized infusion during surgery independent of AAA formation. Circumferential cyclic strain values remained stable in proximal and distal aortic areas of all rats and mice over 28 days (data not shown). Blood pressure data acquired in live AngII* apoE* ^*−/−*^ mice showed that on average they became hypertensive as early as day 3 after implantation, although this increase was not statistically significant (*P* < 0.1). The tail cuff technique is inherently noisy, meaning that the increase in blood pressure did not reach significance of *P* < 0.05. However, longitudinal measurements (Figure 2(b) inset) provided evidence of continued hypertension in the animals over the course of the study.

### 3.3. Mean Blood Flow Velocity Decreases in Both AAA Models and Recirculates in Suprarenal AAAs

**Figure 3:**
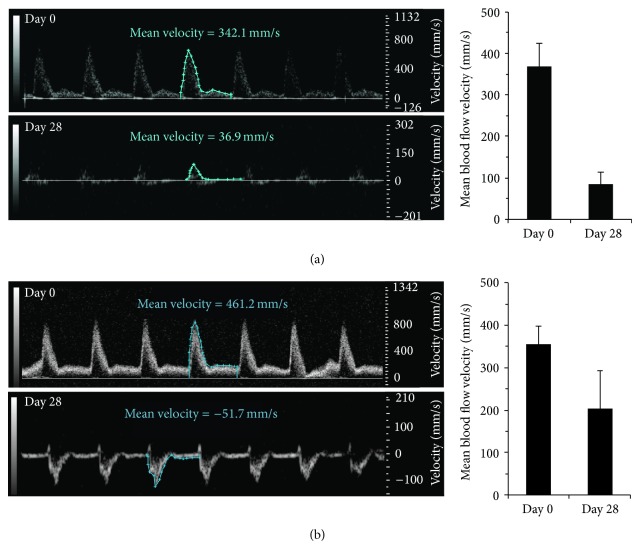
Mean aortic blood flow velocity decreases after 28 days. Representative velocity waveforms on long-axis PW Doppler acquisitions (days 0 and 28) and corresponding measurements of mean blood flow velocities at site of AAA in high-concentration-elastase rats (a) and AngII* apoE* ^*−/−*^ mice (b).

With progression of disease in the high-concentration-elastase rats, we found that mean blood flow velocity progressively decreased over time (Figure 3(a), graph). Overall decreases in this value at areas adjacent to an AAA were visually evident (Supplementary Videos 3 and 4) and measurable (Figure 3(a), velocity waveform plots). We found comparable reductions in AAA mean blood flow velocity (Table 1) at day 28 relative to day 0 (76.9 ± 29.1%; *P* < 0.05) and to adjacent healthy regions at day 28 (73.5 ± 27.3%; *P* < 0.001). In low-concentration-elastase rats, we found that the mean blood flow velocity in the perfused region had slightly decreased after 28 days (*P* = 0.0532) (Table 1).

PW Doppler scans on AngII* apoE* ^*−/−*^ mice did not provide a complete dataset. However, we clearly observed a shift to complex aortic flow patterns by days 21 and 28 imaging (Supplementary Video 5). Smaller areas of reduced or recirculating flow were seen adjacent to focal dissection points (Supplementary Video 6). As a result, we measured marked reductions in mean blood flow velocity and a change in flow direction at day 28 relative to day 0 in mice (Figure 3(b), velocity waveforms). We found comparable reductions in AAA mean blood flow velocity at day 28 relative to day 0 (49.6 ± 26.9%; *P* = 0.192) and to adjacent healthy regions at day 28 (52.9 ± 28.9%; *P* < 0.05) (Table 1).

### 3.4. AAAs Exhibit Elastin Degradation and Collagen Deposition by Histological Staining

**Figure 4:**
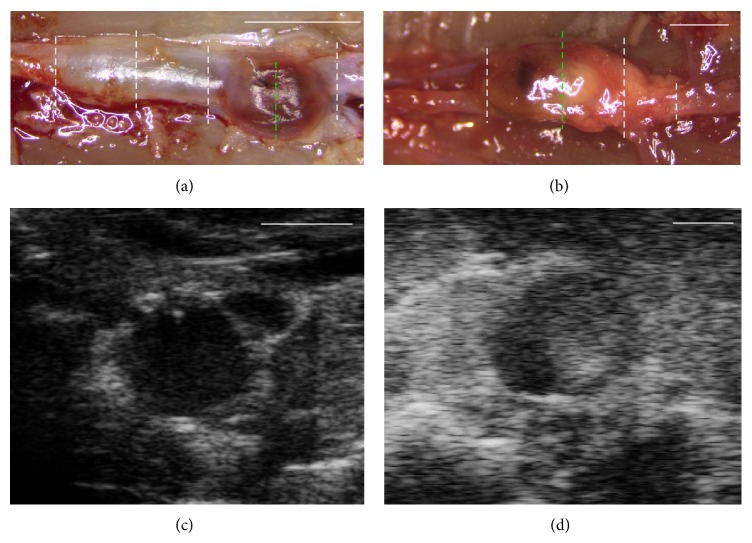
Gross dissection of AAAs shows shape of AAAs detected by B-mode images. End-of-study dissection images of AAAs* in situ* and short-axis B-mode images at day 28 from a high-concentration-elastase rat ((a), (c)) and an AngII* apoE* ^*−/−*^ mouse ((b), (d)). B-mode images taken at the approximate anatomical locations marked by green dashed lines ((a) and (b)). White dashed lines indicate locations selected for histology. Scale bars: 5 mm (a); 2 mm (b); 2 mm (c); and 1 mm (d).

**Figure 5:**
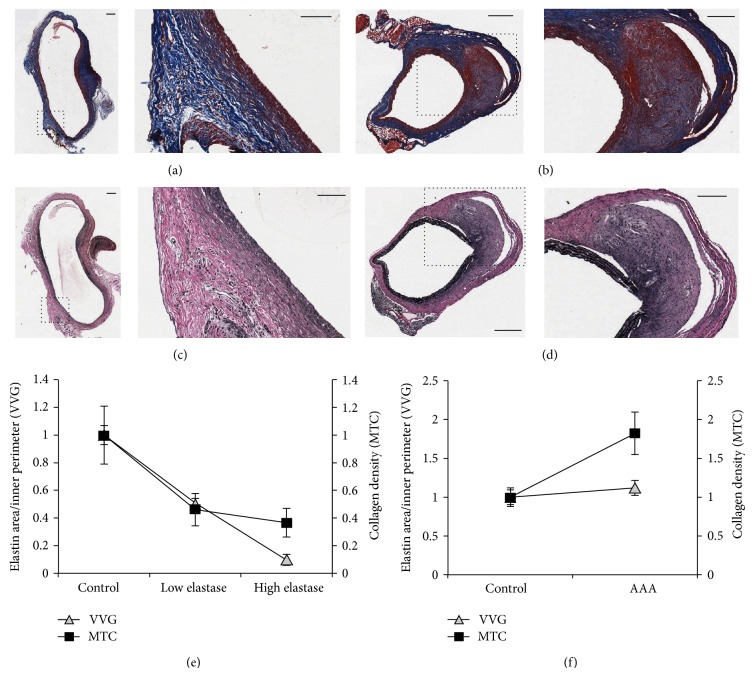
Histological staining reveals qualitative and semiquantitative differences in elastin and collagen content of AAAs. Representative MTC ((a), (b)) and VVG ((c), (d)) images of AAA tissue from a high concentration elastase rat ((a), (c)) and an AngII* apoE* ^*−/−*^ mouse ((b), (d)). Normalized measurements of mean elastin and collagen content ((e), (f)) in high- (*n* = 5) and low- (*n* = 4) concentration-elastase rats (e) and AngII* apoE* ^*−/−*^ mice (*n* = 6) (f). Controls (*n* = 3 in each group) are sections with healthy regions. Scale bars: 200*μ*m at low and 100*μ*m at high magnification. Level of magnification: 8x ((a), (c)) and 2x ((b), (d)) relative to low magnification.

Dissections of rats and mice showed the anticipated shape of AAAs* in situ* as was seen by ultrasound prior to sacrifice (Figures 4(a) and 4(b)). The short-axis B-mode images in Figures 4(c) and 4(d) reveal the inner vessel walls and maximum cross-sectional diameters and areas in each AAA model prior to dissection. MTC staining for collagen (Figures 5(a) and 5(b)) and VVG staining for elastin (Figures 5(c) and 5(d)) revealed changes in the distribution and overall content of these proteins in the AAA wall. The most noticeable changes are the degradation and loss of elastin bands in high-concentration-elastase rats and significant collagen deposition in the AngII* apoE* ^*−/−*^ mice. Collagen accumulates as disorganized collagen fibers, as is seen with fibrosis, and fails to support the aortic wall architecture [24]. We found that reductions in elastin content were statistically significant using high elastase concentration (90 ± 3.9%; *P* = 0.002) relative to healthy regions (Figure 5(e)). Low-concentration-elastase rats exhibited a smaller amount of elastin degradation (49 ± 3.1%; *P* = 0.122) in some of the aortas (images not shown). Additionally, the morphology of collagen changed in both low- and high-concentration-elastase rats, appearing stringier and more detached than healthy collagen fibers. In these areas, we found a statistically significant decrease in the amount of collagen in high-concentration-elastase rats (63 ± 10% reduction; *P* = 0.04). Collagen in low-concentration-elastase rats also decreased (54 ± 11%), albeit not significantly (*P* = 0.13).

AngII* apoE* ^*−/−*^ mice consistently showed collagen deposition in and around transmural hematomas resulting from false lumen formation. We found an 82 ± 27% increase (*P* = 0.0611) in collagen content in these areas relative to healthy regions (Figure 5(f)). Conversely, our measurements of elastin content in proximity to the hematomas showed similar levels to those for healthy regions (*P* = 0.73), as a focal dissection point where elastin had been degraded was not always seen in our sections.

### 3.5. Nonlinear Optical Microscopy Enables Label-Free Imaging of Elastin and Collagen in* Ex Vivo* AAA Tissue

**Figure 6:**
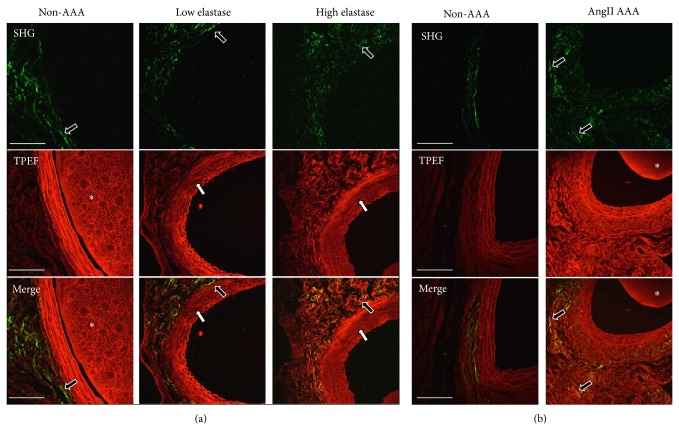
Nonlinear optical microscopy provides a label-free approach for examination of AAA tissue morphology and protein content. SHG (green), TPEF (red), and merged SHG/TPEF nonlinear optical images of representative aortic tissue sections from healthy and AAA regions in low- and high-concentration-elastase rats (a), and AngII* apoE* ^*−/−*^ mice (b). Scale bars: 100*μ*m. Black arrows point to adventitial collagen; white arrows point to degraded elastin bands; and asterisks indicate agarose gel in lumen.

We also tested the use of NLO microscopy to detect elastin and collagen in AAA tissues. We found that AAAs from elastase rats showed changes in TPEF signal according to elastase concentration. Elastin fibers were clearly absent or degraded in elastase perfused aortic tissue (white arrows in Figure 6(a)), similar to the findings seen with VVG staining. In contrast, healthy regions demonstrated strong autofluorescence signal due to the presence of elastin. The emission filter used also detected autofluorescence signal from agarose gel in the aortic lumen (asterisks in Figure 6) and adventitial proteins, likely collagen and fibrin. Adventitial collagen, as detected by SHG signal, was observed in both types of AAAs (black arrows in Figure 6). This signal was visually apparent in and around transmural hematomas in the AngII* apoE* ^*−/−*^ model where collagen deposition is pronounced. In the adventitia of elastase-perfused aortas, a change in overall SHG signal intensity and spatial distribution may also be occurring.

## 4. Discussion

This work demonstrates that we can sensitively detect and monitor biomechanical changes occurring during progression of AAAs in both the elastase and AngII* apoE* ^*−/−*^ models. We showed reductions in both circumferential cyclic strain and mean blood flow velocity within AAAs relative to adjacent healthy regions. We also found substantial elastin fragmentation and collagen deposition with label-free microscopy. Taken together, these results suggest that differences in AAA growth, strain, and blood flow velocity are useful parameters that reveal time-dependent changes in each model.

While aortic diameter measurements are standard for determining maximum aortic expansion in both human [25–27] and small animal [15–18, 28, 29] AAAs, the data from this study suggest that aortic volumes may be a more useful metric for characterizing aneurysm expansion. Indeed, simple diameter measurements from B-mode images depend on the acquisition location and measurement technique. Aortic volumes, on the other hand, should be less dependent on location and technique as the entire aorta can be acquired in one three-dimensional volume. This technique can also account for changes in the length of a growing AAA not exhibiting accompanying increases in diameter. Similar to the volumetric ultrasound images others have collected [30, 31], we observed an increase in the length of AngII* apoE* ^*−/−*^ AAAs. The average AAA volume-to-length ratios in our AngII* apoE* ^*−/−*^ mice (mean ± SE; 2.8 ± 0.3 mm^2^ at day 28) compare well with a previous report using MRI (mean ± SD; 2.5 ± 0.6 mm^2^ at day 28 [21]). Likewise, these values for the high concentration elastase rats (8.7 ± 1.6 mm^2^ at day 28) compare well to elastase-perfused mice (1.1 ± 0.2 mm^2^ at day 28 [21]) when accounting for animal weight (rats are roughly 10 times bigger than mice). We also observed several mice and rats in this study where the diameter measurements plateaued, but the volume/length ratio continued to increase (Figure 1). These results suggest that in some cases the aneurysms were lengthening without significant diameter expansion. A recent article also used three-dimensional ultrasound but for* ex vivo* measurements of AAAs from AngII* apoE* ^*−/−*^ mice [32]. These volumes, however, were segmented over a short 2 mm length in all cases, making a direct comparison to our measurements with variable lengths difficult. Future studies to compare volumes of the same AAAs* in vivo* and* ex vivo* by ultrasound may be warranted, as the changes in the overall size and shape of AAAs following transcardial perfusion are not fully known.

With both AAA models, we noted that circumferential cyclic strain sometimes decreased independently of vessel expansion. In AngII* apoE* ^*−/−*^ mice, four animals had reductions in circumferential cyclic strain (>33%) but normal effective maximum diameters (<150% baseline) at day 3. Three animals showed abrupt aortic expansion in parallel with large reductions in circumferential cyclic strain (>75%). Consistent with this finding is blood pressure data, which shows sustained hypertension starting at day 3. This is expected with AngII infusion, leading to a shift in the loading response up the stress-strain curve. At these early time points we would not expect there to be substantial vessel remodeling. Further studies with greater numbers of mice are needed to improve our understanding of the relationship between vessel expansion and circumferential cyclic strain reductions in AngII-induced AAAs. For rats infused with low-concentration elastase, we observed reductions in circumferential cyclic strain despite negligible increases in aortic diameter or volume over 28 days (Table 1). This may be due to pressure-induced vessel damage, ligation of branching vessels, or other effects of the surgical procedure. Two groups have previously carried out studies to track changes in circumferential strain values in the aortas of AngII* apoE* ^*−/−*^ mice [19, 21]. Favreau et al. [19] calculated linear or Cauchy strain values using ultrasound and found a roughly 40% reduction in cyclic strain in the supraceliac aorta at day 3 (similar to the circumferential cyclic strain reduction of −49.7 ± 11.9% we observed). Ideally, the nonlinear strain components accounted for by circumferential cyclic strain should not be neglected since strain values up to 25% cannot be considered small [22]. Goergen et al. [21] calculated circumferential cyclic strain values from magnetic resonance angiography (MRA) images of elastase (19.4 ± 2.5%) and AngII* apoE* ^*−/−*^ (20.8 ± 4.2%) mice at baseline in relative agreement with our values (15.2 ± 1.2% in high- and low-concentration-elastase rats and 12.6 ± 1.4% in AngII* apoE* ^*−/−*^ mice). However, we observed comparatively larger reductions by day 28. High-concentration-elastase rats in our study showed an 83.1 ± 31.1% reduction, while their elastase mice had a 44.8 ± 0.1% decrease. AngII* apoE* ^*−/−*^ mice in this study showed an 80.2 ± 21.6% reduction, while the other paper showed a 52 ± 16.3% decrease in this same model. This difference may be due to the higher spatial resolution and fundamental contrast differences between the Vevo2100 ultrasound system and MRA using a 4.7 T magnet. As MRA highlights unsaturated spins or nuclei from flowing blood, its disadvantage lies in accurately visualizing solid aneurysm regions where blood flow is not present. For the AngII* apoE* ^*−/−*^ mice, MRA showed maximal reductions in circumferential cyclic strain and increases in aortic diameter coincided in the days 7 to 14 interval [21]. This delay compared to our findings of earlier reductions in strain may be attributable to the fact that we primed the pumps in physiological saline solution for at least 12 hours prior to implantation.

Many previous efforts have used qualitative assessments of VVG- and MTC-stained vessels to confirm elastin breakage and collagen deposition [33, 34]. We focused on a quantitative approach that would provide a more robust comparison between suprarenal AAAs and infrarenal AAAs from high- and low-concentration-elastase rats. Goergen et al. previously quantified differences in elastin content of VVG-stained aortas [21] and found that the anterior wall in elastase-perfused mice showed significantly greater elastin loss than the posterior wall in the same region. We implemented a similar code with the addition of vessel wall collagen analysis in order to determine the magnitude of change in AAA areas relative to adjacent healthy areas.

The use of NLO microscopy (SHG and TPEF) was included in these studies as it is a powerful label-free technique that can be used to study vascular diseases. Changes in the content and morphology of elastin and collagen are clearly tied with inflammatory and proteolytic effects occurring in AAA progression. Future studies could benefit from three-dimensional mapping and quantification of elastin and collagen fibers. Cui et al. demonstrated the use of multiphoton microscopy for volumetric analysis of these macromolecules in the aorta and skin of a murine Marfan model [35], while Haskett et al. determined vessel fiber alignment after mechanical testing of AAAs [36]. Le et al. originally measured the emission spectra of collagen and elastin in atherosclerotic Ossabaw pig arteries using a similar microscopy set-up [23]. Taken together, these previous studies and our work suggest that a label-free imaging approach has several advantages over standard histological staining and microscopy. Primarily, it obviates the need for tissue processing and chemical staining. The tissue can be imaged in a near-native state and variability in tissue stain application is no longer an issue. It also permits analysis of thicker tissue sections than is feasible with confocal microscopy. Similar to how volumetric ultrasound measurements of AAAs provide more information than simple two-dimensional measures, the use of volumetric quantification from thicker tissue specimens could also be useful when imaging* ex vivo* sections.

There are several limitations in the present study. First, the presence of abdominal gas in animals can disrupt ultrasound imaging. If not resolved within a few minutes, we changed the orientation of the transducer relative to the body to minimize this interference. However, this reorientation sometimes limited our ability to visualize all regions of the aorta and to detect accurate blood flow patterns. Additionally, the Doppler measurements in the study had large variations. This was likely due to variations in the Doppler angle when acquiring PW images. Thus, tilting and rotating the animals could be done in order to optimize the Doppler angle for long-axis PW and color Doppler measurements in the aorta where the vessel often runs parallel to the transducer.

## 5. Conclusions

We carried out longitudinal studies* in vivo* in order to image and measure several parameters relevant to AAA development. Elastase-perfused rats and AngII* apoE* ^*−/−*^ mice exhibited large reductions in circumferential cyclic strain and mean blood flow velocity over 28 days. These parameters had temporal differences in each model that were not necessarily correlated with increases in effective maximum diameter or AAA volume. The results suggest that vessel strain and blood flow are important metrics and may be indicative of underlying disease processes in AAAs.

We used small animal ultrasound for this study, as it offered us rapid imaging speed and versatility in imaging acquisitions and was relatively inexpensive compared to other tomographic imaging. Furthermore, semiquantitative histology and nonlinear optical microscopy allowed us to further characterize our findings* ex vivo*. We feel that quantifying macromolecular changes in elastin and collagen is helpful for sensitively characterizing AAAs from both the elastase and AngII* apoE* ^*−/−*^ models. Future studies could use these methods to investigate mechanisms in pathogenesis and treatment effects relevant to AAAs and other cardiovascular diseases.

## Supplementary Material

The videos referenced in the text are available online as Supplementary Material (). Video 1 is a cine loop seen in long-axis with Mmode tracing of an infrarenal AAA in a high concentration elastase rat at day 28. Video 2 is a cine loop seen in long-axis with M-mode tracing of a suprarenal AAA in an AngII *apoE-/-* mouse at day 28. Video 3 is a long-axis Color Doppler cine loop of a fusiform AAA in a high concentration elastase rat at day 28. Video 4 is a long-axis Color Doppler cine loop in a more bulbous area of the perfused region in a high concentration elastase rat at day 28. Video 5 is a long-axis Color Doppler cine loop of a saccular AAA in an AngII *apoE-/-* mouse at day 28. Video 6 is a short-axis Color Doppler cine loop of a suprarenal AAA in an area with false lumen inflow in an AngII *apoE-/-* mouse.

## Figures and Tables

**Table 1:** Summary of values measured at days 0 and 28 in low- and high-concentration-elastase rats and AngII *apoE* ^−/−^ mice.

	Elastase rats		Elastase rats		AngII *apoE* ^−/−^ mice	
	(0.44–0.48 U/mL)		(25 U/mL)		(1000 ng·kg^−1^·min^−1^)	
	(mean ± SE (*n* = 6))		(mean ± SE (*n* = 5))		(mean ± SE (*n* = 7))	
	Day 0	Day 28	Day 0	Day 28	Day 0	Day 28
Effective maximum diameter (mm)	1.48 ± 0.04	1.55 ± 0.05	1.43 ± 0.03	4.02 ± 0.29^*^	1.01 ± 0.05	2.27 ± 0.15^*^
Aortic maximum diameter (mm)	1.15 ± 0.09	1.46 ± 0.14	1.05 ± 0.03	3.85 ± 0.49^*^	0.95 ± 0.05	2.06 ± 0.13^*^
Volume/length (mm^2^)	1.72 ± 0.06	2.06 ± 0.29	1.41 ± 0.06	8.65 ± 1.57^*^	0.70 ± 0.08	2.76 ± 0.26^*^
Length (mm)	6.92 ± 0.40		7.88 ± 0.83		7.07 ± 0.65	
False lumen volume (mm^3^)	NA	NA	NA	NA	0	11.1 ± 1.57^*^
Circumferential cyclic strain (%)	17.28 ± 1.34	6.18 ± 0.72^*^	12.67 ± 1.39	2.14 ± 0.27^*^	12.6 ± 1.4	2.5 ± 0.61^*^
Mean BFV (perfused region/AAA) (mm/s)	NA	230 ± 28.4	NA	85.3 ± 29.6°	NA	189 ± 99.9^∧^
Mean BFV (healthy region) (mm/s)	318 ± 11.3		370 ± 55.4	322 ± 41.9^*^	374 ± 44.7	401 ± 57.3

Statistical significance shown for *α* = 0.05: ^*^versus the vessel measurement at day 0; ^∧^versus healthy region measurement at day 28; °versus healthy region measurement at day 0.

Data are given as mean ± SE. BFV: blood flow velocity; NA: not applicable.
